# Loss of chondroitin sulfate proteoglycan sulfation allows delayed sympathetic reinnervation after cardiac ischemia–reperfusion

**DOI:** 10.14814/phy2.15702

**Published:** 2023-05-24

**Authors:** Matthew R. Blake, Diana C. Parrish, Melanie A. Staffenson, Morgan A. Johnson, William R. Woodward, Beth A. Habecker

**Affiliations:** ^1^ Department of Chemical Physiology and Biochemistry Oregon Health and Science University Portland Oregon USA; ^2^ Department of Neurology Oregon Health and Science University Portland Oregon USA

## Abstract

Sympathetic nerve loss in the heart predicts the risk of ventricular arrhythmias after myocardial infarction (MI) in patients. Sympathetic denervation after cardiac ischemia–reperfusion is sustained by matrix components chondroitin sulfate proteoglycans (CSPGs) in the cardiac scar. We showed that 4,6‐sulfation of CSPGs was critical for preventing nerve growth into the scar. Promoting early reinnervation with therapeutics reduces arrhythmias during the first 2 weeks after MI, but the longer‐term consequences of restoring innervation are unknown. Therefore, we asked if the beneficial effects of early reinnervation were sustained. We compared cardiac function and arrhythmia susceptibility 40 days after MI in mice treated on Days 3–10 with vehicle or with intracellular sigma peptide to restore innervation. Surprisingly, both groups had normal innervation density in the cardiac scar 40 days after MI, indicating delayed reinnervation of the infarct in vehicle‐treated mice. That coincided with similar cardiac function and arrhythmia susceptibility in the two groups. We investigated the mechanism allowing delayed reinnervation of the cardiac scar. We found that CSPG 4,6‐sulfation, which is elevated early after ischemia–reperfusion, was reduced to control levels allowing reinnervation of the infarct. Thus, remodeling of extracellular matrix weeks after injury leads to remodeling of sympathetic neurons in the heart.

## 1 INTRODUCTION

Coronary artery disease is a leading cause of mortality in the United States, and a critical contributor is the approximately 1.2 million Americans who suffer a myocardial infarction (MI) each year. Those who survive an MI have increased risk of sudden cardiac arrest (Pouleur et al., 2010; Rubart & Zipes, 2005; Solomon et al., 2005), and the amount of sympathetic nerve loss in the myocardium predicts the risk of ventricular arrhythmias that often follow MI (Boogers et al., 2010; Fallavollita et al., 2014; Nishisato et al., 2010; Vaseghi et al., 2014).

Sympathetic denervation after ischemia–reperfusion is sustained by chondroitin sulfate proteoglycans (CSPGs) in the cardiac scar (Gardner & Habecker, 2013). CSPGs are components of the extracellular matrix (Gilbert et al., 2005) that bind protein tyrosine phosphatase receptor sigma (PTPσ) on sympathetic neurons to suppress nerve regeneration (Gardner et al., 2015; Gardner & Habecker, 2013). Removing PTPσ or disrupting PTPσ‐TrkA interactions using intracellular sigma peptide (ISP) or small molecules restores sympathetic nerve regeneration into the cardiac scar after MI, and prevents isoproterenol induced arrhythmias (Blake, Gardner, et al., 2022;Gardner et al., 2015; Sepe et al., 2022). We recently showed that 4,6‐sulfation of CSPG side chains increased after ischemia–reperfusion, and that disrupting CSPG 4,6‐tandem sulfation was sufficient to allow nerve regeneration into the scar and decrease arrhythmia susceptibility (Blake, Parrish, et al., 2022). Promoting early reinnervation reduces arrhythmias during the first few weeks after MI (Blake, Parrish, et al., 2022; Gardner et al., 2015; Sepe et al., 2022), but the longer‐term consequences of restoring sympathetic transmission throughout the left ventricle are unknown.

We wondered whether the beneficial effects of restoring normal sympathetic innervation throughout the left ventricle were sustained, given the pathological effects of excessive sympathetic transmission in the heart (Eisenhofer et al., 1996; Herring et al., 2019; Lopez‐Sendon et al., 2004; Meredith et al., 1991). To answer that question, we assessed cardiac function and arrhythmia susceptibility 40 days after MI in mice treated with vehicle or with ISP, which modulates PTPσ in sympathetic neurons to promote reinnervation through CSPGs (Gardner et al., 2015; Sepe et al., 2022). To our surprise, vehicle‐ and ISP‐treated animals both had normal innervation density in the cardiac scar 40 days after ischemia–reperfusion. This coincided with similar cardiac function and arrhythmia susceptibility 40 days after injury. We investigated the mechanism allowing delayed reinnervation of the cardiac scar, and found that CSPG 4,6‐sulfation, which is elevated in the first 2 weeks after ischemia–reperfusion (Blake, Parrish, et al., 2022), was reduced to control levels allowing reinnervation of the infarct. Thus, remodeling of extracellular matrix weeks after injury leads to remodeling of sympathetic neurons in the heart.

## 2 METHODS

### 2.1 Animals

C57BL/6J mice obtained from Jackson Laboratories West were used for all experiments. All mice were kept on a 12 h:12 h light–dark cycle with ad libitum access to food and water. Age and gender‐matched male and female mice 12–18 weeks old were used for all experiments. Animals were treated on Days 3–10 after surgery via IP injection with either ISP (10 μM) or Vehicle (5% DMSO/saline) (Gardner et al., 2015). All procedures were approved by the OHSU Institutional Animal Care and Use Committee and comply with the Guide for the Care and Use of Laboratory Animals published by the National Academies Press (8th edition). Animals were euthanized by decapitation while under deep isoflurane (4%–5%) anesthesia. Reporting of animal experiments conforms to the principles and regulations for animal experimental reporting and ethics in the ARRIVE guidelines (Kilkenny et al., 2010).

### 2.2 Myocardial ischemia–reperfusion procedure

Anesthesia was induced with 4% isoflurane and maintained with 2% isoflurane. The left anterior descending coronary artery was reversibly ligated for 40 min and then reperfused by release of the ligature (Gardner & Habecker, 2013; Parrish et al., 2010). Occlusion was confirmed by sustained S‐T wave elevation and regional cyanosis. Reperfusion was confirmed by the return of color to the ventricle distal to the ligation and reperfusion arrhythmia. Core body temperature was monitored by a rectal probe and maintained at 37°C, and a two‐lead electrocardiogram was monitored.

### 2.3 Left ventricle tissue collection/processing

Hearts were collected 40 days after ischemia–reperfusion surgery unless otherwise noted. Animals were euthanized and hearts were excised and rinsed in saline solution to remove as much blood as possible. For western blot or norepinephrine analysis, ventricles were placed into a brain slicer on ice and sectioned into 1 mm sections. Scar tissue was dissected from the slices, snap frozen in liquid nitrogen, and stored at −80°C for batch processing. For immunohistochemistry, hearts were fixed for 1 h in 4% paraformaldehyde, rinsed again in PBS, and cryoprotected in 30% sucrose (Li et al., 2004).

### 2.4 Cardiac tissue immunoblotting

Heart tissue was pulverized in a glass douncer in NP40 lysis buffer (50 mM Tris [pH 8.0], 150 mM NaCl, 2 mM EDTA, 10 mM NaF, 10% glycerol, and 1% NP‐40) containing complete protease inhibitor cocktail (Roche), phosphatase inhibitor cocktails 2 and 3 (Sigma). Lysates sat on ice for 30 min with intermittent vortexing. Lysates were centrifuged (13k rpm, 10 min, 4°C). 50–100 μg of protein lysate was treated for 6–8 h with chondroitinase ABC (100 μU/mL; R&D Systems) before being resolved on 4%–12% bis‐tris gel (BioRad Criterion XT) by SDS/PAGE, transferred to nitrocellulose membrane (GE Life Sciences), and blocked in 5% nonfat milk (Blake, Parrish, et al., 2022). Samples were probed with mouse anti‐chondroitin‐4‐sulfate (1:1000; Millipore MAB2030), mouse anti‐chondroitin‐6‐sulfate (1:1000; Millipore MAB2035), rabbit anti‐NG2/CSPG4 (1:1000; Millipore AB5320), or rabbit anti‐CHST15 (1:1000; Proteintech 14298‐1‐AP). Blots were washed, incubated with goat anti‐mouse or anti‐rabbit HRP (1:10,000; Thermo 32430 & 32460, respectively), and bound antibody detected by chemiluminescence (Thermo). Protein expression was quantified with ImageJ densitometry and normalized to total protein as measured by Ponceau staining.

### 2.5 Immunohistochemistry

Transverse 12 μm sections of myocardium were cut and mounted onto charged slides. To reduce autofluorescence sections were rinse 3 × 10 min in 10 mg/mL sodium borohydride and rinsed for 3 × 10 min in PBS. The slides were then blocked in 2% BSA, 0.3% Triton X‐100 in PBS at room temperature for 1 h. Sections were then incubated with rabbit anti‐TH (1:1000; Millipore, AB152) overnight. The following day the slides were incubated with goat anti‐rabbit Alexa‐Fluor 488 (1:1000; Thermo A32731) for 1.5 h and rinsed in PBS. To further reduce autofluorescence, slides were incubated in 10 mM CuSO_4_ (diluted in 50 mM ammonium acetate) for 30 min. Slides were rinsed 3 × 10 min in PBS before mounting in 1:1 glycerol:PBS and visualized by fluorescence microscopy. Threshold image analysis was described previously (Lorentz et al., 2010) but briefly, the threshold function in ImageJ was used to generate black and white images discriminating TH + nerves for six sections spanning the infarct/scar or peri‐infarct region from each heart. Percent area TH + fiber density (20× field of view) was quantified within the infarct and the area adjacent to the infarct (peri‐infarct).

### 2.6 Quantification of cardiac scar size

Scar tissue is notably lacking autofluorescence, enabling easy identification of the scar (Gardner et al., 2015). Images were acquired with a Keyence BZ‐X 800 microscope at 2× magnification and analyzed using ImageJ freehand selection tool. Left ventricle (LV) and cardiac scar were outlined and measured. The percent area of cardiac scar was determined by calculating (cardiac scar area/LV area) × 100. The scar was imaged in at least six sections sampled across different levels of the infarct.

### 2.7 Echocardiography

Cardiac function was assessed using echocardiography. High‐frequency fundamental imaging (Vevo 2100) was performed between 25 and 40 MHz (Lorentz et al., 2010; Sepe et al., 2022). Mice were sedated with inhaled isoflurane (1.0%–1.5%). Images were obtained in the parasternal long‐axis plane and parasternal short‐axis planes at the midpapillary level. LV function was assessed by measurement of LV end‐diastolic, end‐systolic area (short axis) and end‐diastolic, end‐systolic length (long axis). Stroke volume was determined using the left ventricular outflow tract area and time‐velocity integral on pulsed‐wave Doppler.

### 2.8 Arrhythmia assessment

Anesthesia was induced with 4% isoflurane and maintained with 2% isoflurane. Surface electrodes were used to monitor ECG, with animals maintained at 37°C throughout the analysis. ECG and temperature data were monitored using Powerlab LabChart software (AD Instruments). A 30‐min baseline was used to assess spontaneous arrhythmias prior to administration of β‐agonist Isoproterenol (50 μg) and caffeine (100 μg) as described previously (Blake, Parrish, et al., 2022; Wang et al., 2014). Arrhythmias were measured for 30 min following drug administration and scored on a scale of 0–4 according to the modified Lambeth conventions (Curtis et al., 2013). Individual animals received a single score based on the most severe arrhythmia observed. 0 indicates no arrhythmia. 1 indicates one to two premature ventricular contractions (PVCs) followed by normal sinus rhythm of at least two beats. 2 indicates bigeminy (one PVC followed by one normal sinus beat, repeating for four or more continuous cycles) or salvo (three to five PVCs in a row). 3 indicates non‐sustained ventricular tachycardia defined as six or more PVCs in a row lasting less than 30 s. 4 indicates sustained VT (>30 s) or Torsades de Pointes.

### 2.9 Statistics

Unpaired *t*‐test was used for comparisons of just two samples, and when standard deviations were unequal Welch's correction was used. For nonparametric analyses the Mann–Whitney test was used. For experiments comparing different treatment groups and a second variable (such as region of myocardium) two‐way ANOVA was carried out using Tukey's multiple comparison test. All statistical analyses were carried out using Prism 9.

## 3 RESULTS

### 3.1 Sympathetic reinnervation of the cardiac scar 40 days after MI regardless of treatment

**FIGURE 1:**
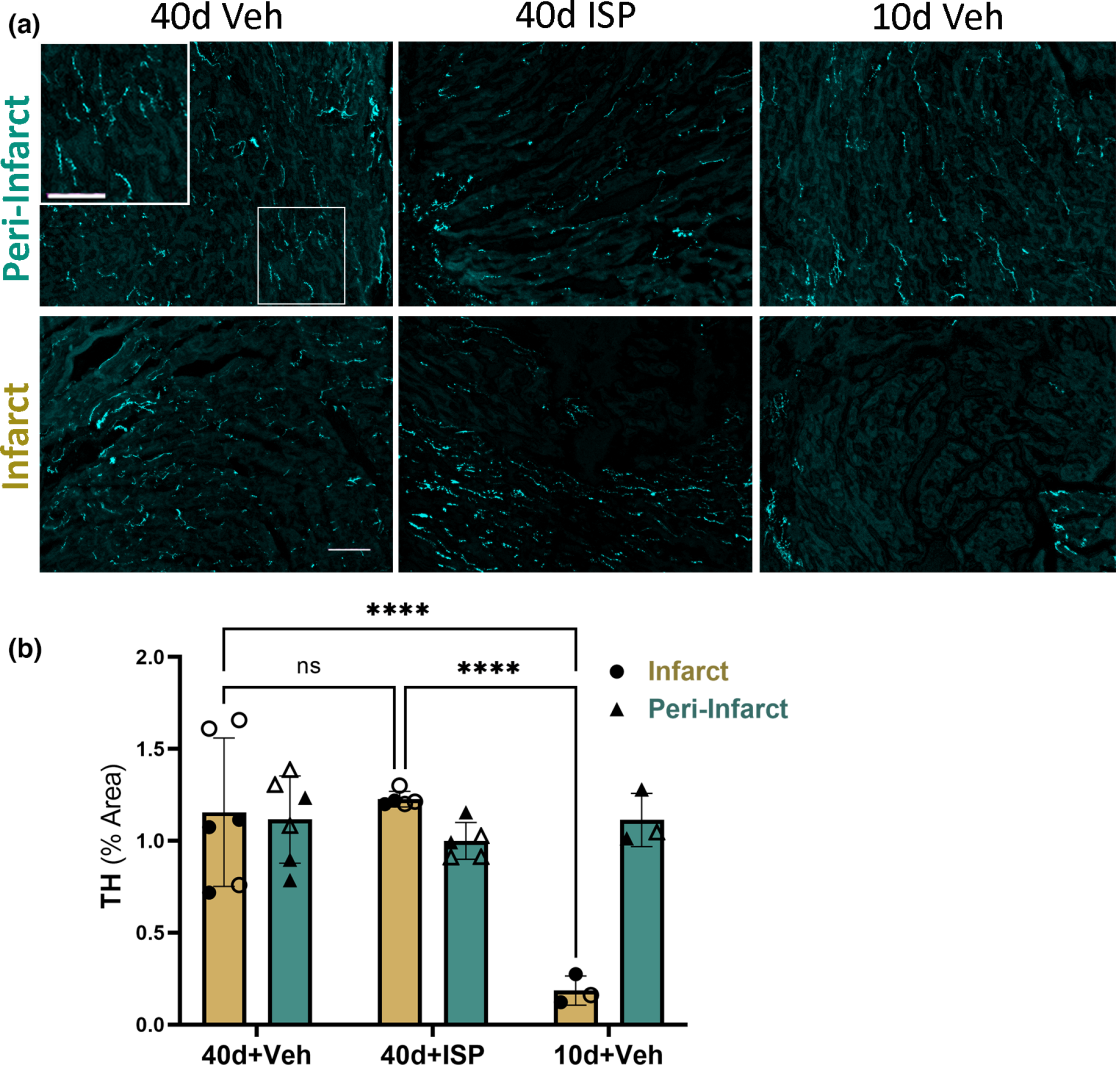
Sympathetic reinnervation of the cardiac scar 40 days after MI. Immunohistochemistry for tyrosine hydroxylase (cyan) identified sympathetic neurons in sections from hearts obtained 40 or 10 days after MI. (a) Example images from Veh‐treated hearts 40 days after MI, ISP‐treated hearts 40 days after MI, and Veh‐treated hearts 10 days after MI. (b) Quantification of TH+ innervation density expressed as % area, *n* = 6 40d + veh (3F, 3 M), *n* = 5 40d + ISP (3F, 2 M), and *n* = 3 10d (1F, 2 M); open symbols = female, closed symbols = male. Two‐way ANOVA, Tukey's post‐test to compare all groups, select comparisons shown, ns, not significant, ****p‐value<0.0001. Scale bar 100 μm. Inset scale bar 50 μm.

The infarct that develops after ischemia–reperfusion lacks significant reinnervation until at least 3 weeks after injury in the absence of PTPσ deletion or administration of a therapeutic to allow nerve growth into the scar (Blake, Parrish, et al., 2022; Gardner et al., 2015; Gardner & Habecker, 2013; Sepe et al., 2022). We analyzed sympathetic innervation density 40 days after ischemia–reperfusion to determine if the reinnervation of the infarct promoted by ISP was sustained. We found that innervation density was normal throughout the left ventricle, including the infarct, in ISP‐treated animals (Figure 1). To our surprise, however, vehicle‐treated animals also exhibited normal innervation density in the infarct 40 days after injury. This differed from a group of vehicle‐treated control mice that were analyzed 10 days after MI that were processed at the same time. Those mice exhibited the expected denervation of the infarct with vehicle treatment and reinnervation with ISP that we have observed previously (Gardner et al., 2015; Sepe et al., 2022).

### 3.2 Cardiac function is similar in vehicle‐ and ISP‐treated animals 40 days after MI

**FIGURE 2:**
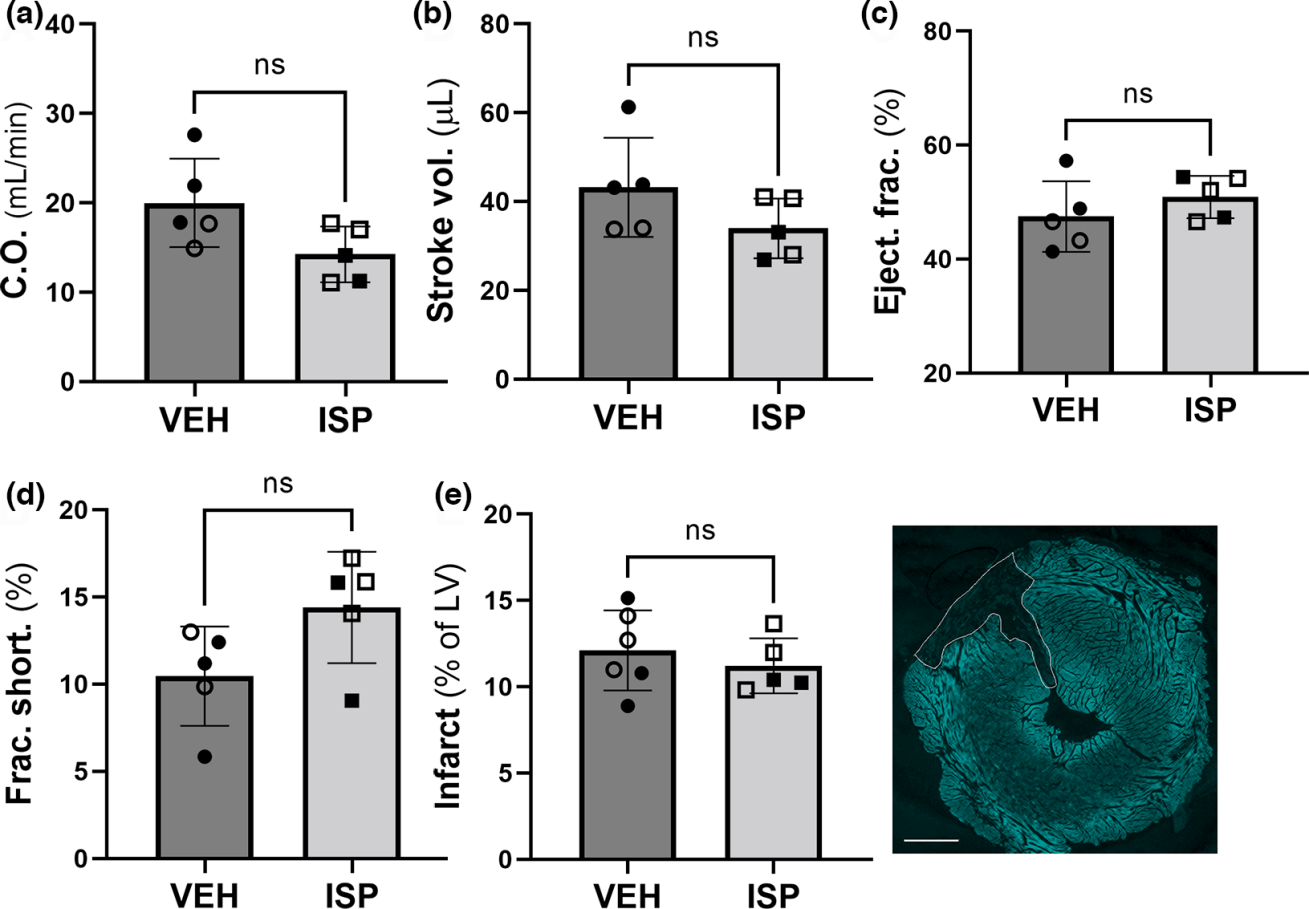
Early reinnervation does not alter cardiac function or infarct size 40 days after MI. Functional parameters were similar in vehicle (VEH)‐ and ISP‐treated mice 40 days after MI. (a) Cardiac output (C.O.), (b) stroke volume, (c) ejection fraction, and (d) fractional shortening. Data are Mean ± SD, *n* = 5 animals/group (VEH 2F, 3 M; ISP 3F, 2 M); open symbols = female, closed symbols = male; unpaired *t*‐test. (e) Infarct size is similar 40 days after MI in VEH‐ or ISP‐treated hearts. Mean ± SD, VEH *n* = 6 (3F, 3 M), ISP *n* = 5 (3F, 2 M); open symbols = female, closed symbols = male; unpaired *t*‐test. Sample image of LV with infarct outlined in a white line. Scale bar = 500 μm.

Cardiac function was measured by echocardiography 40 days after ischemia–reperfusion surgery. ISP‐treated animals had similar cardiac output (Figure 2a), stroke volume (Figure 2b), ejection fraction (Figure 2c), and fractional shortening (Figure 2d) when compared with vehicle‐treated animals. Likewise, LV mass was not different between the two groups: Veh 120 ± 25 mg; ISP 102 ± 11 mg, Mean ± SD (*n* = 5; VEH 2F, 3 M; ISP 3F, 2 M), and infarct size was not significantly different between vehicle‐ and ISP‐treated groups (Figure 2e). Together these data suggest that restoring normal sympathetic neurotransmission to the infarcted myocardium during the first week after injury does not lead to later changes in infarct size or cardiac function.

### 3.3 Arrhythmia susceptibility is similar in vehicle‐ and ISP‐treated animals 40 days after MI

**FIGURE 3:**
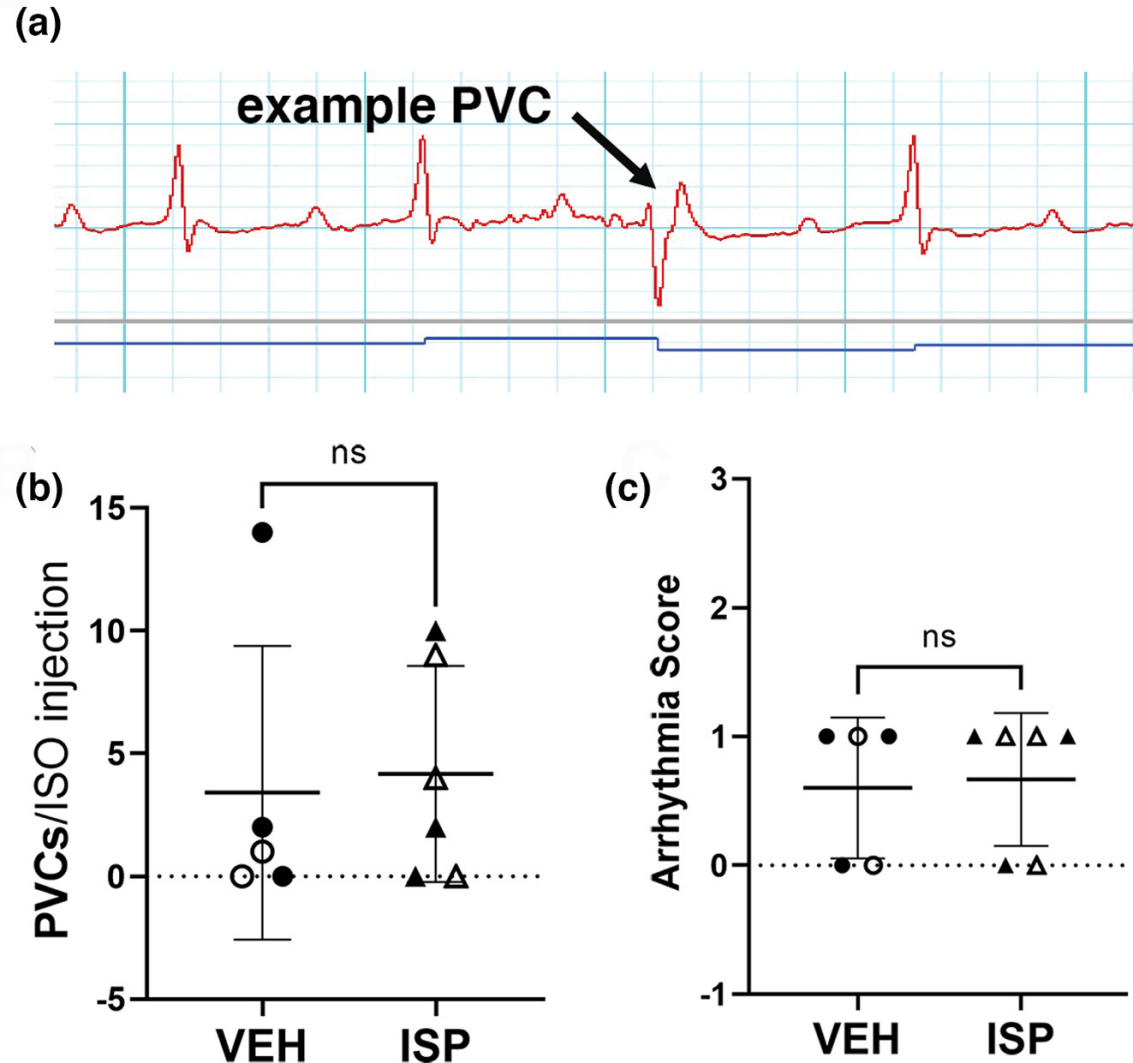
Arrhythmia susceptibility is similar 40 days after MI with vehicle or ISP treatment (a) Example PVC after administration of Isoproterenol + caffeine in a vehicle‐treated animal 40 days after MI. (b) Number of PVCs occurring after injection of Iso/caffeine. Data are mean ± SD, *n* = 5–6 (VEH 2F, 3 M; ISP 3F, 3 M); open symbols = female, closed symbols = male. Unpaired *t*‐test with Welch's correction; ns‐ not significant; *p* = 0.8. (c) Arrhythmia scores after injection of Iso/caffeine. Arrhythmia scores are based on the most severe arrhythmia observed in each heart (0 = no PVCs, 1 = single PVCs) See methods for additional details. *n* = 5–6 (VEH 2F, 3 M; ISP 3F, 3 M); open symbols = female, closed symbols = male; Mann–Whitney test; ns‐ not significant; *p* = 0.9.

Early reinnervation significantly decreases isoproterenol‐induced arrhythmias 10–21 days after MI, when the borderzone and infarct are largely denervated in the absence of therapeutic intervention (Blake, Parrish, et al., 2022; Gardner et al., 2015; Sepe et al., 2022). We assessed arrhythmia susceptibility 40 days after MI in vehicle‐ and ISP‐treated animals, administering isoproterenol and caffeine to trigger arrhythmias. We found similar arrhythmia susceptibility in both groups, consistent with similar innervation densities in the borderzone and scar. Arrhythmias were quantified by two methods which gave the same results. The first method was to count all premature ventricular complexes (PVCs) that occurred within 30 min of the isoproterenol/caffeine injection. The second method was to score the most severe arrhythmia in each mouse ranging from 0 (no PVCs) to 4 (sustained VT) as described in the methods section. Individual PVCs were the most severe arrhythmia type observed in any of the mice, and the number of PVCs was not significantly different between the two groups (Figure 3). This contrasts with studies carried out at 14 days using the same ECG testing conditions, when hearts with denervated infarcts exhibited more severe types of arrhythmias (Blake, Parrish, et al., 2022).

### 3.4 Reduced levels of CSPG sulfation in the cardiac scar 40 days after MI

**FIGURE 4:**
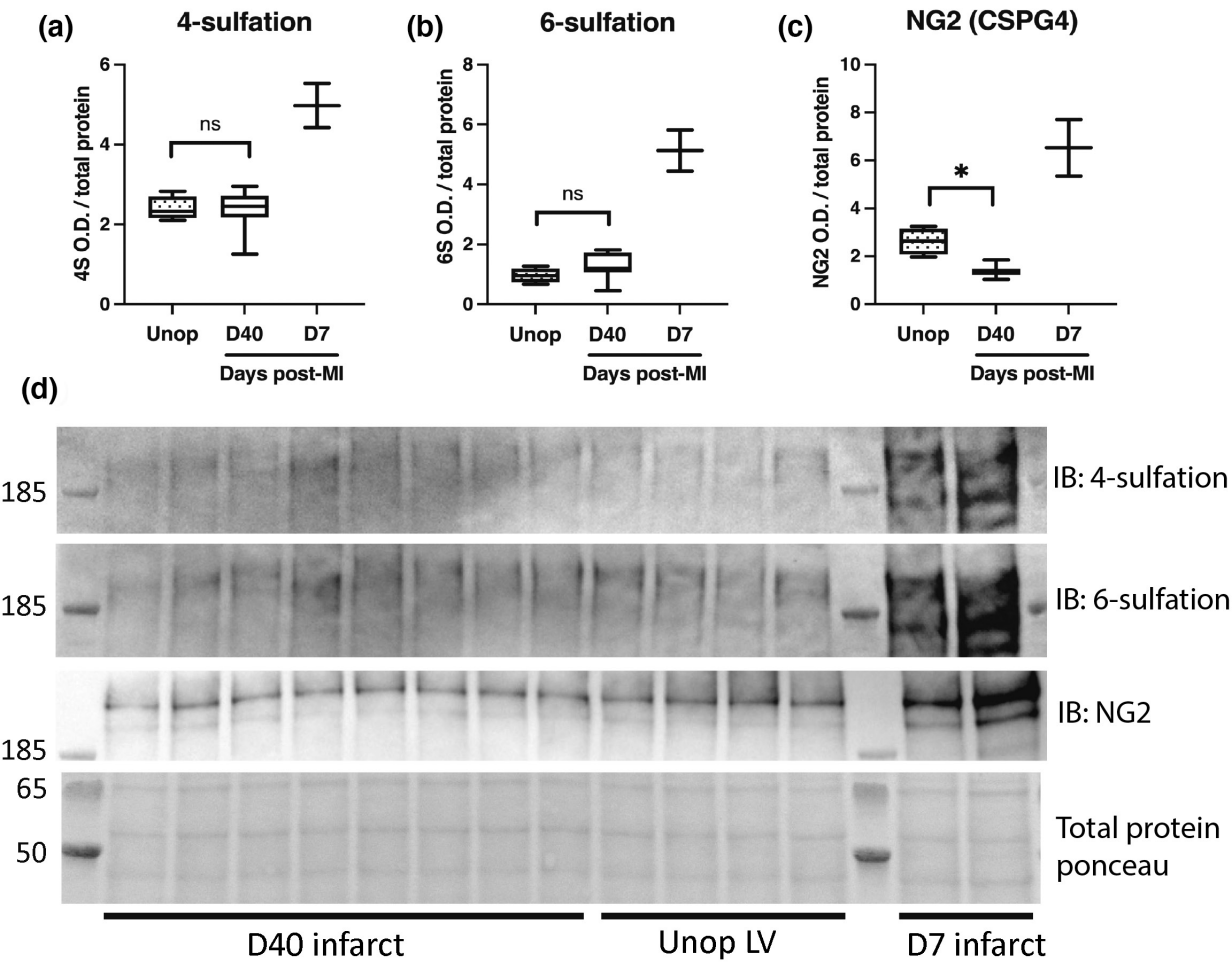
Chondroitin 4,6‐sulfation is reduced to control levels 40 days after MI. Western blot quantification of (a) 4‐sulfation (4S CS‐GAGs), (b) 6‐sulfation (6S CS‐GAGs), and (c) NG2 core protein and in the cardiac scar 40 days after MI compared to unoperated LV tissue. For visual comparison, two samples in the blots are from D7 post‐MI when sulfation levels are high in the infarct; these were quantified but not used for statistical comparison due to sample size. *n* = 8 animals for D40 post‐MI vehicle treated (4F, 4 M) and *n* = 4 animals for unoperated left‐ventricle group (2F, 2 M); unpaired *t*‐test (Welch's correction), ns, not significant, *p < 0.05. (d) Western blot images of a–c.

Our previous work demonstrated that 4,6‐sulfation of CSPG side chains increased after MI and was a critical inhibitor of sympathetic axon extension into the infarct (Blake, Parrish, et al., 2022). In light of the sympathetic nerve growth into the cardiac scar that occurred between Days 21 and 40 after ischemia–reperfusion, we asked whether CSPG sulfation had changed during that time. Using antibodies specific to chondroitin 4‐ and 6‐sulfation we found that sulfation at both positions in the cardiac scar 40 days post‐MI had returned to the level in unoperated control left ventricle (Figure 4). 4,6 tandem sulfated GAGs (4S,6S) are produced by a 4S dependent chondroitin‐6‐sulfotransferase, CHST15 (Miller & Hsieh‐Wilson, 2015) which increases in the first weeks after MI (Blake, Parrish, et al., 2022), but returns to control levels at 40 days. Optical density values normalized to protein: Unop 0.9 ± 0.5 mean ± SD, *n* = 4 (2F, 2 M); 40 days 1.0 ± 0.4 mean ± SD, *n* = 8 (4F, 4 M), *p* = 0.55. Interestingly, the CSPG core protein NG2 in cardiac scar was suppressed below the level present in unoperated LV 40 days after MI (Figure 4). These data suggest that the production and sulfation of CSPGs that is induced after ischemia–reperfusion (Blake, Parrish, et al., 2022) ultimately returns to baseline levels after several weeks, allowing sympathetic nerves to grow into the cardiac scar.

## 4 DISCUSSION

Reperfusion is standard of care for a coronary artery occlusion, but the infarct that forms after ischemia–reperfusion contains CPSGs which prevent regeneration of sympathetic nerves into the scar (Gardner et al., 2015; Gardner & Habecker, 2013). Regional sympathetic nerve loss after MI predicts the risk of ventricular arrhythmias in patients (Boogers et al., 2010; Fallavollita et al., 2014; Nishisato et al., 2010). We identified several approaches to promote sympathetic nerve regeneration into the denervated scar after MI, and found that restoring innervation to the borderzone and infarct decreases isoproterenol‐induced arrhythmias (Blake, Gardner, et al., 2022; Gardner et al., 2015; Sepe et al., 2022). Here we asked if the beneficial effects of restoring normal sympathetic innervation throughout the left ventricle were sustained. The key findings of this study are as follows: (1) 40 days after ischemia–reperfusion, sympathetic nerve density was normal in the infarct of all animals regardless of treatment group. (2) Cardiac function and arrhythmia susceptibility were indistinguishable in mice with early or late reinnervation of the infarct. (3) Sulfated CSPGs, which are induced by ischemia–reperfusion and prevent reinnervation, have returned to control levels 40 days after injury. This loss of sulfated CSPGs allows sympathetic nerve growth into the infarct (Blake, Parrish, et al., 2022).

The most surprising finding of this study was that sympathetic nerves grew into the cardiac scar in vehicle‐treated mice. Damaged myocardium produces high levels of nerve growth factor (NGF), a crucial trophic factor stimulating sympathetic axon outgrowth, within hours of ischemia–reperfusion (Hiltunen et al., 2001). NGF expression stays elevated for an extended time after MI (Meloni et al., 2010; Zhou et al., 2004), produced by multiple cell types including macrophages (Hasan et al., 2006), but in the first 3 weeks after ischemia–reperfusion axonal growth is blocked by CSPGs (Gardner et al., 2015; Gardner & Habecker, 2013). Thus, we reasoned that the delayed reinnervation was due to the loss of inhibitory CSPG signaling. We recently showed that CSPG 4,6‐sulfation was important for preventing nerve regeneration in the heart (Blake, Parrish, et al., 2022), so we asked if sulfation was reduced 40 days after MI. We found that CSPG 4,6‐sulfation was reduced to the level seen in undamaged myocardium. Additionally, the major CSPG core protein in the heart, NG2, which is elevated in the first 2 weeks after MI (Blake, Parrish, et al., 2022), was reduced to below the level observed in unoperated myocardium. Our earlier work showed that changes in the expression of multiple enzymes was responsible for sustaining the tandem sulfation of CSPGs in the cardiac scar (Blake, Parrish, et al., 2022), including increased 4S dependent chondroitin‐6‐sulfotransferase CHST15. The level of CHST1 had normalized back to control levels 40 days after injury, consistent with the loss of sulfation. These data suggest resolution of the injury response includes remodeling of extracellular matrix in a manner that allows nerve regeneration into the scar.

Inflammatory cells are involved in the cardiac remodeling process (Dobaczewski et al., 2006), and it is not known if immune cells modulate the production or sulfation of CSPGs in the cardiac scar. Although the impact of immune cells on CSPGs is unclear, in spinal cord injury and multiple sclerosis the presence of CSPGs seems to increase inflammation (Dyck et al., 2018; Francos‐Quijorna et al., 2022; Stephenson et al., 2018). Treating mice with ISP after spinal cord injury shifts the immune response toward a more reparative phenotype and enhances restoration of function, potentially through direct effects on immune cells (Dyck et al., 2018; Francos‐Quijorna et al., 2022). We found that treating mice with ISP after MI restores sympathetic innervation and shifts the inflammatory response toward a more reparative phenotype (Sepe et al., 2022), but the increase in reparative leukocytes within the heart did not alter infarct size or cardiac output 14 or 40 days after MI.

Another parameter that was similar between ISP‐ and vehicle‐treated mice was arrhythmia susceptibility. Individual PVCs were the most severe arrhythmia observed in any of the mice, and the number of PVCs was not significantly different between the two groups. This contrasts with studies carried out at 14 days using the same ECG testing conditions, when hearts with denervated infarcts exhibited numerous PVCs as well as more severe types of arrhythmias including non‐sustained VT (Blake, Parrish, et al., 2022). Thus, reinnervation decreases arrhythmia susceptibility.

There are some weaknesses in this study. We do not have the full time course of reinnervation or enzyme expression between Days 21 and 40. We also do not have arrhythmia data from conscious mice since the ECG implants are not well tolerated for this length of study. Thus we used both caffeine and isoproterenol to trigger arrhythmias. Extracellular matrix remodeling follows a similar trajectory after ischemia–reperfusion in the mouse and dog, but the time course is significantly faster in the mouse (Dobaczewski et al., 2006). It remains unclear how the time course of CSPG expression and sulfation in the mouse compares to larger animals and human hearts, and will be an important issue to address in the future.

In summary, we asked if early sympathetic reinnervation after MI had longer term effects on cardiac function or arrhythmia susceptibility. We found that it did not, and that ongoing remodeling of the extracellular matrix ultimately allows sympathetic axon extension into infarcted myocardium.

## FUNDING INFORMATION

This work was supported by NIH F31HL152490 (MRB), AHA 20PRE35210768 (MRB), and NIH R01 HL093056 (BAH).

## CONFLICT OF INTEREST STATEMENT

B.A. Habecker is co‐inventor of technology (ISP) that is used in this research, and that OHSU has licensed to NervGen Pharma Corp. This potential conflict of interest has been reviewed and managed by OHSU.

## ETHICS STATEMENT

All vertebrate animal experiments were performed in accordance with the Animal Welfare Act and the Office of Laboratory Animal Welfare regulations, NIH, USA. The OHSU Institutional Animal Care and Use Committee approved all animal protocols.
